# Complete mitochondrial genome of the mantis shrimp, *Chorisquilla orientalis* Hwang, Ahyong, & Kim, 2018 (Stomatopoda: Protosquillidae)

**DOI:** 10.1080/23802359.2020.1840939

**Published:** 2020-12-24

**Authors:** Hee-seung Hwang, Jiyeong Shin, Jongwoo Jung

**Affiliations:** a Research Institute of EcoScience, Ewha Womans University, Seoul, Korea; b The Division of EcoCreative, Ewha Womans University, Seoul, Korea; c Department of Science Education, Ewha Womans University, Seoul, Korea

**Keywords:** *Chorisquilla orientalis*, Crustacea, mitochondrial DNA, mitochondrial genome, Protosquillidae

## Abstract

In this study, we determined the mitochondrial genome of a stomatopod, *Chorisquilla orientalis*, collected from Korean waters. The complete mitochondrial genome comprised 15,880 bp, encoding 13 proteins, 22 transfer RNAs, 2 ribosomal RNAs genes, and a non-coding A + T rich region. The overall base composition in the heavy strand was A: 35.5%, G: 12.4%, C: 18.3%, and T: 33.7%, with a G + C content of 30.8%. Phylogenetic analysis showed that *C. orientalis* belonged to the families Gonodactylidae and Takuidae in the same clade, and to the superfamily Gonodactyloidea within Stomatopoda. This is the first record of the complete mitochondrial genome sequence of the family Protosquillidae.

Mantis shrimps or stomatopods have large and powerful raptorial appendages that can be used for ‘smashing’ or ‘spearing’ (Caldwell and Dingle 1976). More than 480 stomatopod species have been described worldwide (Ahyong 2001, 2004), they play important roles in numerous marine ecosystems, due to their enormous biomass and nutritional position as both prey and predator (Geary et al. 1991; Ahyong et al. 2013). Of them, all protosquillids have a smashing-type of raptorial claw and include more than 35 species in 6 genera have been reported (Ahyong 2010). In this study, we provide the first complete mitochondrial genome sequence of a Protosquillid species, *Chorisquilla orientalis* Hwang, Ahyong, & Kim.

The specimen was collected by scuba diving from the subtidal zone of Dokdo Island, South Korea (geographic location: 37°14′34.9′′N, 131°52′08.6′′E) on 16 July 2018, and was preserved in 95% ethyl alcohol until mitogenome analysis. The voucher specimen was deposited at the Research Institute of EcoScience, Ewha Womans University (EWNHMAR769). Total DNA was extracted from leg muscle tissue using DNeasy Blood and Tissue kit (Qiagen, Hilden, Germany) and the DNA library was prepared using TruseqNano DNA Prep Kit (Illumina, San Diego, CA). The mitochondrial DNA (mtDNA) was sequenced using Illumina Novaseq 6000 system. MITObim (Hahn et al. 2013) was used for the assembly of the complete mitochondrial genome, which was then annotated using MITOS (Bernt et al. 2013).

The length of the mitogenome of *C. orientalis* is 15,880 bp encoding 13 proteins, 22 transfer RNAs, 2 ribosomal RNAs, and a non-coding A + T-rich control region. For the 13 protein-coding genes (PCGs), the most common shared start codon was ATG in *COX2*, *COX3*, *ATP6*, *NAD3*, *NAD4*, *NAD4L*, and *CYTB*, followed by ATT in *NAD2* and *NAD6*. The start codon for *COX1* was ACG, which has also been reported in the mtDNA of malacostraca (Cook 2005; Liu and Cui 2010), while *NA1* and *NAD5* started with ATA, and *ATP8* with an ATC. The stop codon in all PCGs was TAA, except for *COX2* and *NAD6* that ended with the incomplete stop codons AAT and CCT, respectively. Incomplete stop codons have been identified in several PCGs of all the stomatopod mitochondrial genomes published to date, and this has been attributed to excessive polyadenylation (Ojala et al. 1980, 1981). The overall base composition in the heavy strand was A: 35.5%, G: 12.4%, C: 18.3%, and T: 33.7%, with a G + C content of 30.8%. The *LrRNA* and *SrRNA* genes in *C. orientalis* had 1353 bp and 838 bp, respectively. The size of the 22 transfer RNAs encoded ranged from 64 to 72 nucleotides. The putative control region was of 947 bp and was located after the transfer RNA-Val and the *SrRNA*.

The phylogenetic tree was constructed based on sequences of 13 PCGs identified by the maximum likelihood (ML) method using MEGA X (Kumar et al. 2018). The GTR + G + I model was identified as the best-fit model for the data, using ModelFinder (Kalyaanamoorthy et al. 2017) with 1,000 bootstrap replicates.

To confirm the phylogenetic position of *C. orientalis*, four squilloid species and two gonodactyloid were compared with our *C. orientalis* (MT672286), using a lysiosquilloid species as an outgroup. The analysis showed that *C. orientalis* was grouped with *Gonodactylus chiragra* of Gonodactylidae and *Taku spinosocarinatus* of Takuidae, in one clade with high bootstrap value, and it belonged to the superfamily Gonodactyloidea within Stomatopoda (Figure 1).

**Figure 1.**
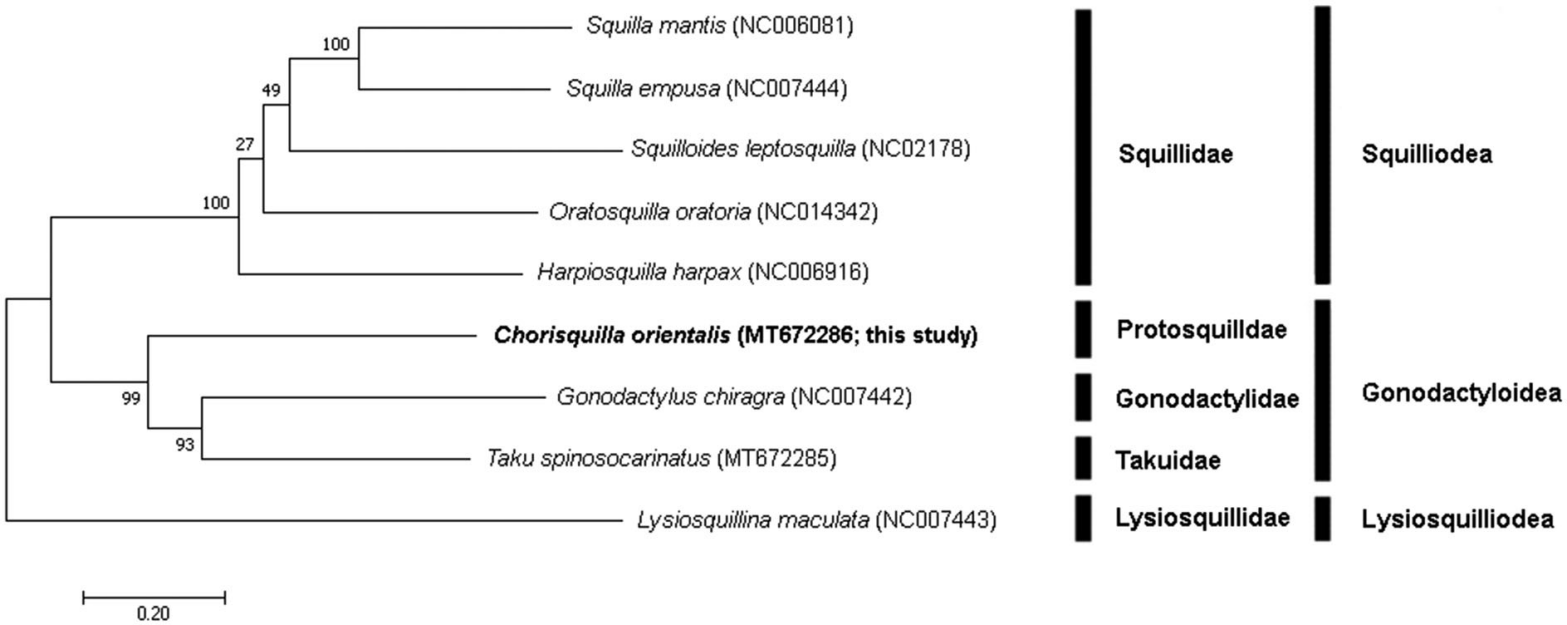
Phylogenetic tree of complete mitochondrial genomes from nine stomatopods (*Oratosquilla oratoria* (NC014342), *Gonodactylus chiragra* (NC007442), *Harpiosquilla harpax* (NC006916), *Squilla empusa* (NC007444), *Squilla mantis* (NC006081) *Lysiosquillina maculata* (NC007443), *Taku spinosocarinatus* (MT672285), and *Chorisquilla orientalis* (MT672286)) constructed using maximum likelihood (ML) method.

This is the first report of the complete mitogenome sequence of the family Protosquillidae. The results of this study provide useful information for further phylogenetic and evolutionary studies on stomatopod members in the future.

## Data Availability

The genome sequence data that support the findings of this study are openly available in GenBank of NCBI at https://www.ncbi.nlm.nih.gov under the accession no. MT672286. The associated BioProject, SRA, and Bio-Sample numbers are PRJNA663208, SRR12649330, and SAMN16122871, respectively. The data that support the findings of this study are also openly available in Mendeley Data at http://dx.doi.org/10.17632/khwyfjmt7g.1
